# GM1 Ganglioside as a Disease-Modifying Therapeutic for Parkinson’s Disease: A Multi-Functional Glycosphingolipid That Targets Multiple Parkinson’s Disease-Relevant Pathogenic Mechanisms

**DOI:** 10.3390/ijms24119183

**Published:** 2023-05-24

**Authors:** Jay S. Schneider

**Affiliations:** Department of Pathology and Genomic Medicine, Thomas Jefferson University, Philadelphia, PA 19107, USA; jay.schneider@jefferson.edu

**Keywords:** GM1 ganglioside, Parkinson’s disease, disease modification

## Abstract

Parkinson’s disease (PD) is a progressive neurodegenerative disorder affecting millions of patients worldwide. Many therapeutics are available for treating PD symptoms but there is no disease-modifying therapeutic that has been unequivocally shown to slow or stop the progression of the disease. There are several factors contributing to the failure of many putative disease-modifying agents in clinical trials and these include the choice of patients and clinical trial designs for disease modification trials. Perhaps more important, however, is the choice of therapeutic, which for the most part, has not taken into account the multiple and complex pathogenic mechanisms and processes involved in PD. This paper discusses some of the factors contributing to the lack of success in PD disease-modification trials, which have mostly investigated therapeutics with a singular mechanism of action directed at one of the many PD pathogenic processes, and suggests that an alternative strategy for success may be to employ multi-functional therapeutics that target multiple PD-relevant pathogenic mechanisms. Evidence is presented that the multi-functional glycosphingolipid GM1 ganglioside may be just such a therapeutic.

## 1. Introduction

Parkinson’s disease (PD) is the second most common neurodegenerative disease of mid-to-late life, with approximately 1 in every 200 persons in the U.S. aged 60–69, 1 in 100 persons over 70, and 1 in every 35 persons over 80 estimated to have PD [1]. From 1990 to 2015, the number of people with PD worldwide increased 118% to over 6 million [2], global estimates in 2019 showed over 8.5 million individuals with PD [3], and by 2040, the number of people with PD globally could exceed 12–17 million [4]. Thus, with a significant and increasing global prevalence of PD, and its enormous impact on patients, families, and society as a whole, it is important to develop therapies that will slow the progression of this disease to allow patients to enjoy a longer amount of time with a higher quality of life. While currently available pharmacotherapies can at least transiently improve various aspects of functional impairment in patients with PD, the disease continues to progress, and functional abilities continue to deteriorate over time.

At present, no treatments have been shown to significantly and unequivocally slow the progression of PD and thus none have been approved as disease-modifying therapies for this disease. With a large amount of the pre-clinical and clinical research being conducted in the PD field, why is this the case? Why have we not been able to produce an effective disease-modifying therapy for PD? Why have the potential disease-modifying agents that have reached the clinical trial stage failed? The reasons are many and involve a variety of factors that include practical issues regarding the choice of patients for disease modification trials and the best trial designs to detect disease modification (see [5] for review of these issues), but perhaps more importantly, the factor playing a significant role in the failure to produce a disease-modifying therapy for PD has been the choice of therapeutics that have been evaluated and the underlying mechanism(s) of action targeted by these therapeutics.

Basic principles of drug discovery dictate identifying a disease with a high unmet medical need and then identifying a druggable target associated with that disease on which to assess the efficacy of test compounds. A drug candidate must then demonstrate high selectivity for engaging the target of interest with little to no off-target effects, and show an appropriate physiological response to target engagement. Typically, only those compounds with high affinity binding to the target, high selectivity, and demonstrated efficacy are chosen as lead compounds, and if proven to be safe, taken to the clinic. For the most part, these are the types of compounds that have been used in clinical trials as potential disease-modifying therapeutics for PD. However, when considering a potential disease-modifying therapeutic for PD, the choice of an agent that has a singular target or is unifunctional may not be the best strategy. As will be discussed in the remainder of this paper, the focus on unifunctional drugs has been an unproductive approach to finding a clinically effective disease-modifying therapy for a complex neurological disease such as PD, a disease that has multiple contributing pathogenic factors. This paper will briefly discuss the complex pathogenic mechanisms that contribute to neurodegeneration in PD, the multi-functional properties of GM1 ganglioside, and present the rationale for and evidence supporting the further development and potential of GM1 ganglioside as a multi-functional disease-modifying agent for PD.

## 2. Multiple and Complex Pathogenic Mechanisms in Parkinson’s Disease

**Figure 1:**
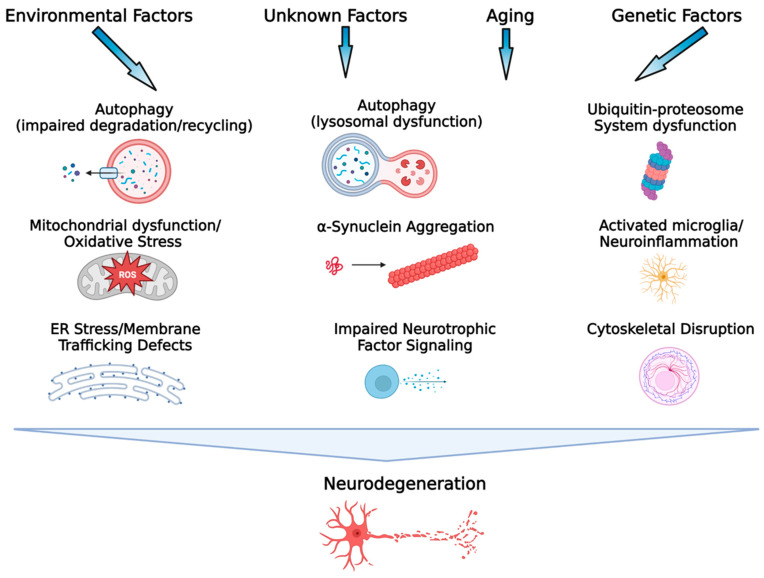
Key processes associated with the etiopathogenesis of PD. Environmental exposures/lifestyle, genetic factors, the aging process, gene–environment interactions, and potentially unknown factors contribute to the etiology of PD. A variety of mechanisms associated with cellular dysfunction, alone and interactively, drive the neurodegenerative process in PD. It is this heterogeneity and complexity of the pathogenic mechanisms involved in PD that have made development of a disease-modifying therapeutic challenging. (Figure created with BioRender.com).

It has been known for some time that the pathogenic and pathophysiological mechanisms contributing to the initiation and progression of brain pathology in PD are multi-factorial and complex (see [6] for review; Figure 1). However, several key pathogenic mechanisms have been identified and these include: dysfunction of mitochondria [7] and defects in mitochondrial respiration, which contributes to increased oxidative stress and oxidative damage, along with enhanced calcium conductance in substantia nigra pars compacta (SNc) dopamine (DA) neurons [6,8]; defects in the ubiquitin–proteasome system involved in the non-lysosomal degradation and clearance of damaged proteins, which leads to potentially toxic accumulation and aggregation of misfolded/damaged proteins [9]; abnormal phosphorylation and aggregation of toxic α-synuclein [10,11,12]; dysfunction of the autophagy–lysosome pathway (ALP), resulting in reduced elimination of potentially excess, defective, and toxic proteins [13,14,15]; endoplasmic reticulum (ER) dysfunction and ER stress that can result in disruptions in cell homeostasis through a wide range of mechanisms including disruption of calcium homeostasis, abnormal synthesis, glycosylation, and folding of proteins, and abnormal synthesis and transport of membrane proteins, resulting in signaling defects [16,17]; cytoskeletal disruption and microtubule dysfunction resulting in impaired axonal transport, synaptic dysfunction, and neurodegeneration [18]; impaired neurotrophic signaling, particularly for glial-derived neurotrophic factor (GDNF) and brain-derived neurotrophic factors (BDNF), and dysfunction of their respective receptor signaling mechanisms (Ret and TrkB) [19,20]; decreased tyrosine hydroxylase expression and activity and decreased DA synthesis in SNc neurons [21]; decreased expression of *NURR1*, which is necessary for normal function of mature DA neurons [22,23]; and microglial activation and neuroinflammation [24,25]. While the above-mentioned factors are often discussed as significant contributors to the neurodegeneration in PD, in reality, the neurodegeneration in PD is most likely driven by a combination of these factors as well as complex interactive effects between many of them, making the development of an effective disease-modifying agent for PD immensely difficult. As this author has stated previously, since PD is a complex disease involving multiple mechanisms and pathways that lead to cell death, an effective disease-modifying therapy would likely need to influence a wide range of cellular mechanisms and functions implicated in PD in order to successfully interfere with the disease process [26]. This sentiment has also been expressed recently by Lenka and Jankovic [5], “As a clinical trial aimed at a disease modification targets only one of the many pathological substrates of PD, it is possible that other potential substrates keep contributing to the progression of the disease”. Taking this into account, one option, as suggested by Lenka and Jankovic [5], is to aim for a synergistic treatment approach using multiple disease-modifying therapies influencing multiple disease-related pathways. However, this approach is potentially fraught with difficulties, including the decision of which mechanisms/pathways to target as well as how to control for unanticipated interactions and undesired off-target effects of combination therapies, as well as potential regulatory roadblocks that must be overcome for combination products. What is proposed here is an alternative approach that takes advantage of the multi-functionality of the glycosphingolipid GM1 ganglioside.

## 3. GM1 Ganglioside: A Multi-Functional Glycosphingolipid Important for the Development and Function of the Nervous System

**Figure 2:**
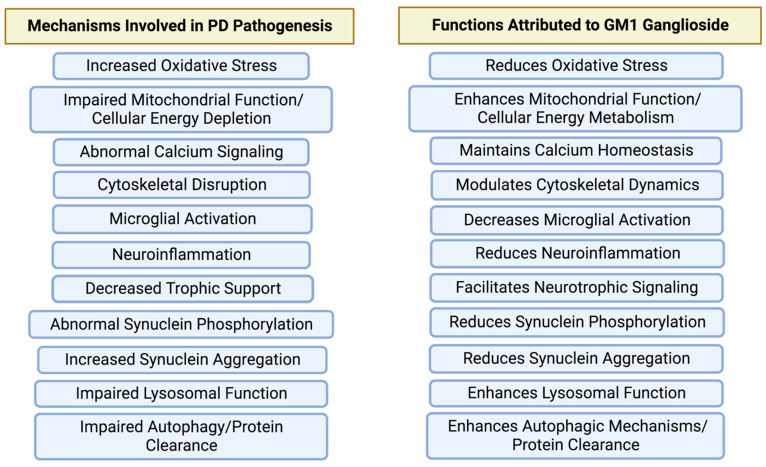
GM1 ganglioside influences multiple target mechanisms relevant to the pathogenesis of PD. While putative disease-modifying therapeutics tested in clinical trials or currently in development typically target one of these mechanisms, GM1 ganglioside targets most of the relevant mechanisms associated with the pathogenesis of PD. (Figure created with BioRender.com).

Gangliosides are sialic-acid-containing glycosphingolipids, of which the major species in brain are a- and b-series gangliosides GM1, GD1a, GD1b, and GT1b, which account for 65–85% of the ganglioside content of the brain [27,28]. All brain gangliosides contribute to the lipid composition of plasma and intracellular membranes, but GM1 ganglioside is of particular interest as it is a main component of membrane-signaling domains (lipid rafts), has been well-studied in developing and mature brains, and has been shown to play particularly important roles in neuronal development, differentiation, neurite outgrowth, and neuron survival, and modulates a variety of cell functions, with particularly important roles in processes of potential relevance to PD (Figure 2). These include: regulating signal transduction [27,28]; ion transport modulation; and neurotrophic signaling (in which GM1 is of particular importance and necessary for membrane insertion and function of TrkA [29], activation of the Ret receptor complex by GDNF through association with the GFRα1/Ret complex [30], and modulation of the activation of TrkB, at least in part through stimulation of BDNF release [31]). Moreover, also related to its neuroprotective and plasticity-related effects is the ability of GM1 to stimulate phosphorylation of ERK1/2 and activation of ERK signaling pathways, increasing expression of p-AKT and p-GSK3β, and reducing expression of pro-apoptotic factors Bcl2 and Caspase-3 [32,33]. 

Studies have also shown an important association between GM1 and α-synuclein. The binding of α-synuclein to GM1 maintains α-synuclein in an alpha-helical, non-aggregating, potentially non-toxic conformation [34,35], and in vivo, GM1 protects against α-synuclein neurotoxicity [36]. GM1 also plays a role in facilitating lysosomal function and autophagic processes. Under conditions of impaired autophagy, GM1, either in vivo or in vitro, increased expression of autophagic markers and activation of autophagy regulatory proteins, enhanced autophagy, and facilitated autophagy-related removal of α-synuclein [37,38,39]. In addition to α-synuclein, other clinically important proteins (LRRK2, Parkin, and PINK1) associate with lipid rafts and co-localize with GM1, suggesting the potential for GM1 to influence cellular functions dependent on these proteins [27,40].

In addition to its important role in signal transduction at the plasma membrane, GM1 is also involved in important intracellular functions. GM1 modulates various ion transport mechanisms and is a particularly robust modulator of Ca^2+^ flux across plasma membranes, playing an important role in the regulation of intracellular Ca^2+^ homeostasis [29]. GM1 also associates with the inner membrane of the nuclear envelope, the ER, and mitochondria where it modulates Ca^2+^ transport and can modulate/enhance mitochondrial function and stimulate cerebral energy metabolism [29,41]. GM1 has also been noted to reduce oxidative stress and lipid peroxidation [42] as well as reduce oxidative stress responses [43]. Early studies with GM1 also demonstrated its anti-excitotoxic effects in vitro and in vivo [44,45,46].

GM1 is also present in non-neuronal cells in brain. For example, GM1 promotes glycolysis in astrocytes, which leads to glucose uptake and lactate release by these cells, that in addition to being an energy substrate can participate in the modification of neuronal excitability, promote neuroprotection, and enhance expression of neuroplasticity-related genes [41]. GM1 also plays a role in microglial function. GM1 on the cell surface mediates the internalization of α-synuclein into microglia [47]. GM1 administration in vivo reduces microglial activation and inhibits the expression of neuroinflammatory genes in an α-synuclein over-expression model of PD [48], thus modulating responses to pro-inflammatory signals and exerting anti-inflammatory effects [49].

While the discussion above is not an exhaustive review of the functions of GM1, it does demonstrate that this multi-functional glycosphingolipid is critical for the normal development and function of the nervous system and plays important roles in maintaining the health and survival of neurons. In the sections below, the importance of GM1 in PD and the rationale for its use as a disease-modifying therapeutic are discussed.

## 4. The Relationship of GM1 Ganglioside to Parkinson’s Disease

The role of GM1 in the pathophysiology of PD has previously been discussed in detail by the author of this review in [26], and others have recently discussed this topic as well [50,51,52]. In the sections below, the current thoughts on the role of GM1 in PD are summarized. 

In consideration of the multiple influences of GM1 on the functional integrity of the nervous system, it is easy to see how the reduced expression of GM1 in the brain may contribute to the neurodegenerative processes in PD. In the human brain, there is a normal age-related decline in GM1 and other gangliosides [53,54], particularly GD1a, a metabolic precursor to and a reservoir for GM1 [55]. As discussed recently by Chowdhury et al. [56], despite the decrease in a-series gangliosides with aging, only a small number of individuals develop PD. These authors hypothesized that individuals that start life with GM1/GD1a levels in the low range of normal may reach a point with advanced age where these ganglioside levels reach a point below the threshold necessary to maintain neuronal viability. It has also been posited previously by this author that a potential pathogenic mechanism that may contribute to the degeneration of DA neurons in PD may involve environmental exposures or some disease process (perhaps related to viral infection) that alters the composition of the ganglioside content of the cell, leading to decreased amounts of GM1 (and GD1a) and increased vulnerability of DA neurons to various accumulating stressors over the lifespan, ultimately leading to sufficient neurodegeneration resulting in the development of PD [26,57].

It is now known that there is a deficiency of GM1 (and other brain gangliosides, particularly GD1a) in the PD SN [58,59] and that this is at least in part related to decreased gene expression in the PD brain for key biosynthetic enzymes involved in the synthesis of a- and b-series gangliosides GM1 and GD1b (B3galt4) and GD1a and GT1b (St3gal2). Using in situ hybridization histochemistry, this author reported decreased gene expression of *B3GALT4* and *ST3GAL2* in residual DAergic neurons in the SN in PD cases compared with the SN of age-matched controls [60], suggesting that dysfunctional ganglioside biosynthesis may result in decreased ganglioside levels. Even if this process results in relatively small reductions in GM1 expression, it may be sufficient to disrupt multiple cellular processes and increase the susceptibility of DA neurons to damage, potentially impairing their ability to respond adequately to potentially damaging internal and external stressors. It is also possible that there is enhanced degradation of GM1 in PD that may contribute to the GM1 deficiency in this disease [61,62]. Interestingly, the deficiency in GM1 may extend beyond the SN in PD. Ledeen and colleagues have shown that there is a deficiency in GM1 in other brain regions in PD besides the SN, as well as a systemic deficiency of GM1 in various non-CNS tissues in PD [51,56,63,64]. 

The picture that emerges is one of PD being a GM1 deficiency disorder. As such, GM1 replacement therapy would seem to be an attractive therapeutic option with the potential to slow the degenerative processes in PD as well as perhaps enhance the functionality of the residual DA system. This is supported by extensive preclinical studies of GM1 in MPTP models of PD in mice and nonhuman primates (reviewed in detail elsewhere [58]) as well as in clinical studies with PD patients. In a double-blind, randomized, placebo-controlled delayed start Phase II study of GM1 in PD [65], 77 subjects with mild/moderate PD were randomly assigned to receive GM1 for 120 weeks (early-start (ES) group) or placebo for 24 weeks followed by the use of GM1 for 96 weeks (delayed-start (DS) group). Washout evaluations occurred at 1 and 2 years after the end of treatment. Seventeen additional subjects who received standard-of-care (comparison group) were followed to obtain information about natural disease progression. At week 24, the ES group had significant improvement in the primary outcome measure (i.e., change in Unified Parkinson’s Disease Rating Scale (UPDRS) motor score). The DS group (as well as the standard-of-care comparison group) showed a worsening of scores during the same period. The ES group showed a sustained benefit on the primary outcome measure compared with the DS group up to week 120 and their UPDRS motor scores remained below those recorded at the study baseline. Both treatment groups fared better than the comparison group subjects but had significant worsening of symptoms during the washout period. This study provided clinical proof of concept data that extended use of GM1 may lead to a lower-than-expected rate of PD symptom progression. It is suggested here that the administration of GM1 replaced a sufficient amount of GM1 lost to the disease to enhance functionality and stabilize/protect at-risk DA neurons. This suggestion is supported by additional findings from a related imaging study that evaluated potential effects of GM1 treatment on the integrity of striatal DA terminals. [^11^C]methylphenidate ([^11^C]MP) positron emission tomography (PET) was used as a measure of the concentration of the DA transporter (DAT) in the striatum [66]. The decline in the binding potential of [^11^C]MP in the striatum of PD patients, a surrogate marker of DA terminal density and disease progression, was measured longitudinally in a subset of patients over the course of the above-mentioned delayed start study. The results of this study showed a significant slowing of the loss of [^11^C]MP binding potential in several striatal regions in GM1-treated subjects and, in some cases, the data suggested an increased binding potential in some striatal regions compared with the baseline. The clinical and imaging results from the delayed start trial of GM1 in PD support a potential disease-modifying effect of GM1 replacement therapy in PD.

## 5. Why Is GM1 Ganglioside a Potentially Effective Disease-Modifying Therapeutic for Parkinson’s Disease?

Disease modification is a major unmet medical need in PD and despite a multi-decade effort at developing a disease-modifying therapy for PD, previous attempts have been mostly unsuccessful [67]. There are many possible factors contributing to the lack of success of potential disease-modifying agents in clinical trials in PD and these include suboptimal selection of study subjects, choice of outcome measures, lack of appropriate bio-markers, and other aspects of clinical trial design [5,67]. However, even with the best possible trial design, clinical success is dependent on the choice of therapeutic, and for PD disease-modifying trials, these have primarily been therapeutics that engage a single, specific target and have for the most part, a single, specific mechanism of action. Based on what is known about the multiple and often interacting pathogenic mechanisms associated with PD, the use of highly focused agents targeting a singular aspect of the PD pathophysiology has been a recipe for failure. Lenka and Jankovic [5] recently summarized key Phase II and later neuroprotective/disease-modifying trials in PD, listing targets, outcome measures, and results, and the reader is directed to their paper for details regarding these various studies. Briefly, key agents (and their presumed mechanism(s) of action) that have been tested (mostly unsuccessfully) for disease modification in PD include: *MAO-B inhibitors* (Selegiline and Rasagiline: also inhibit reuptake of DA and have antioxidant and anti-apoptotic effects); *dopamine agonists* (Pramipexole and Ropinirole: antioxidant/free radical scavenging); *calcium channel blockers* (Isradipine: targets L-type calcium channels linked to selective vulnerability of SNc neurons); *Creatine* (reduces oxidative stress, promotes cellular energy homeostasis, and reduces glutamatergic excitotoxicity); *PPAR-gamma agonist* (Pioglitazone: inhibits microglial activation and production of pro-inflammatory cytokines and nitric acid; enhances mitochondria biogenesis and reduction in oxidative stress); *antioxidants* (Tocopheral: free radical scavenger, antioxidant; *CoQ10* (targets mitochondrial oxidative phosphorylation, antioxidant); *urate enhancers* (Inosine: reduces oxidative stress, antioxidant); *GLP-1 receptor agonist* (Exenatide: promotes mitochondrial biogenesis and has anti-inflammatory and neurotrophic effects); *tyrosine kinase inhibitor* (Nilotinib (c-Abl inhibitor): has multiple potential mechanisms including possible reduction in misfolded α-synuclein, and reduces inflammation); *anti-α-synuclein antibodies* (Prasinezumab: directed against the C-terminus of α-synuclein, binds mostly to aggregated α-synuclein but also to monomeric protein; Cinpanemab: directed against the N-terminus of α-synuclein, binds mostly to aggregated α-synuclein); *statins* (Lovastatin: anti-inflammatory, antioxidant effects, anti-apoptotic effects, possible inhibition of α-synuclein aggregation); and *iron chelators* (Deferiprone: reduces iron-mediated oxidative stress). Many putative disease-modifying agents have focused on calcium channel blockade, influencing oxidative stress pathways, enhancing mitochondrial function and cellular energetics, reducing neuroinflammation, or inhibiting α-synuclein aggregation, while a few have targeted multiples of these pathways/mechanisms. According to a recent review of PD therapies in the clinical trial pipeline [68], there were 54 disease-modifying therapy trials active in 2022, with 28 of those in Phase II and 3 in Phase III. Most of the agents in disease-modifying trials were aimed at familiar targets and/or had familiar mechanisms of action: mitochondria and cellular energy, GLP-1R agonism, antioxidation, kinase inhibition, and agents aimed at aggregated α-synuclein. Some additional targets included the microbiome, GBA, and neurotrophic factors. Whether any of these new or repurposed drugs that are aimed at the same disease-related mechanisms as those in previous unsuccessful disease modification trials will have better success, remains to be seen. 

What differentiates GM1 ganglioside from other putative disease-modifying agents for PD is the number of PD-related pathophysiological mechanisms that it may interact with. As the author of this work has described previously [26], the rationale for GM1 as a neuroprotective/neurorestorative therapy for PD stems from the appreciation that PD is a complex disease involving multiple, interacting, and sometimes overlapping pathogenic mechanisms/pathways that can lead to cell death. An effective disease-modifying therapy would need to influence a wide range of mechanisms involved in multiple critical cellular functions to successfully interfere with the disease process in PD. Almost all of the key pathogenic mechanisms known to be involved in PD are the same cellular mechanisms/pathways known to be influenced by GM1 (Figure 2). It has also recently been suggested by others [5,67,69] that multi-purpose or multi-target therapies may be necessary to achieve disease modification in complex diseases such as PD and Alzheimer’s disease. “As a clinical trial aimed at disease modification targets only one of the many pathological substrates of PD, it is possible that other potential substrates keep contributing to the progression of the disease” [5]. Thus, targeting only one (or a few) possible substrates will not provide meaningful disease modification. While the suggestion has also been made to develop a “synergistic approach” to disease modification in PD using multiple putative disease-modifying therapies as combination therapy to influence multiple disease-related pathways [5], it is suggested here that an alternative approach is to use a single multi-functional agent, such as the multi-functional glycosphingolipid GM1, for which there is a clear preclinical rationale for use in PD and for which there is clinical proof of concept data for disease modification (see [26] for review). 

## 6. The Future of GM1 Ganglioside as a Disease-Modifying Therapeutic for PD

Despite a clear rationale for the use of GM1 in PD, as well as a wealth of preclinical data from multiple laboratories and clinical proof of concept data, the clinical development of GM1 as a disease-modifying therapy for PD has not progressed. The author of this review suggests that there are four factors that have contributed to the lack of progress in the clinical development of GM1: source of GM1, delivery of GM1, lack of a biomarker for GM1, and lack of clarity regarding the extent of target engagement. 

The GM1 ganglioside used to date in most preclinical studies has been extracted and purified from animal brains. Most preclinical studies have used bovine brain-derived GM1 although a few studies have also used porcine brain-derived GM1. The GM1 used in clinical studies has been bovine brain-derived. Long-term use of bovine brain-derived GM1 appeared safe, with no consistent clinically significant changes noted in blood chemistry, hematology, or urinalysis, and no development of immunogenic responses to GM1 with up to 5 years of GM1 use [65,70]. However, there are some concerns regarding the use of animal brain-derived gangliosides in humans. One concern is the transmission of prions that may be in animal brains, but this concern has been shown experimentally to be unsupported [71]. Using bovine brain matter spiked with scrapie-infected brain material, Di Martino showed that in a scaled-down version of the manufacturing process used for the extraction and purification of bovine brain gangliosides, there was no detection of prion protein and no infectivity of the final material. Although there are other possible prion agents that can cause transmissible spongiform encephalopathies, the study by Di Martino suggests that the risks of contaminated brain material making it through the extraction, purification, and final sterilization processes intact are likely minimal, but theoretically possible [71]. 

Another concern regarding the use of animal brain-derived GM1 has to do with the molecular differences between animal GM1 and human GM1. Sialic acid is an integral component of GM1 and there are forms of sialic acid that are only found in humans. N-acetylneuraminic acid (Neu5Ac) is a common form of sialic acid and, for the most part, is the only form found in humans [72]. Alternatively, N-glycolylneuraminic acid (Neu5Gc) is the form of sialic acid common in many animal species, including those from which GM1 has been extracted for preclinical or clinical use, but is not found in humans [72]. Thus, it is theoretically conceivable that animal brain-derived GM1 may contain sialic acid species that could potentially trigger an immunological response, at least in some individuals. 

In order to eliminate the potential concerns from the use of animal brain-derived GM1 and for the continued clinical development of GM1, the availability of synthetically produced human GM1 is necessary. Although small amounts of synthetic GM1 have been produced and used in a preclinical mouse PD model [73], the challenge has been to develop large-scale synthetic processes that would allow for the production of commercially viable quantities of synthetic GM1. A recent paper by Yu et al. [74] reports an improved process for the chemo-enzymatic synthesis of GM1 containing the human Neu5Ac that could potentially be used for large-scale production of GM1 ganglioside, although this has not yet been achieved.

In addition to the sourcing issue, the delivery of GM1 for clinical use has been problematic. In some clinical applications, GM1 has been delivered intravenously, although this route of administration is tenable only for relatively acute administration indications [75]. In the PD clinical trials to date, GM1 was administered by subcutaneous injection, with two injections administered per day and a volume of 2.0 mL delivered with each injection [65]. Not surprisingly, the most prevalent adverse events associated with these studies were injection site reactions [65]. In addition to the obvious drawbacks associated with relatively large volume, long-term subcutaneous administration of GM1, there is the important additional factor of the limited ability of GM1 to be transported across the blood–brain barrier [76,77], necessitating administration of relatively high volumes of GM1 solution by peripheral injection [65]. However, even with the limitations on dosing presented by the available formulations of GM1 and the subcutaneous route of delivery, it is possible that due to at least a partially dysfunctional blood–brain barrier, as has been described in PD [78,79], more GM1 likely entered the brain than would have been expected under conditions of an intact blood–brain barrier, and a sufficient and clinically relevant amount of GM1 was delivered to the brain. As even a small deficiency in GM1 in cells can disrupt homeostatic balance [51], it is not unreasonable to suggest that replacement of even a relatively small amount of GM1 may be therapeutically effective. Nonetheless, for GM1 to eventually be adopted as a viable therapeutic for PD, an alternative route of delivery needs to be used. Kumbale et al. [80] were the first to demonstrate that GM1 could be delivered to the cerebrospinal fluid (CSF) via the olfactory pathway and that higher amounts of GM1 were detected in the CSF following administration via the olfactory pathway than by intravenous administration. More recently, Itokazu et al. [81] showed that intranasal infusion of GM1 in a transgenic mouse α-synuclein model reduced intracellular α-synuclein levels and enhanced tyrosine hydroxylase expression in the SN, supporting the potential use of intranasal delivery of GM1 as a potentially viable alternative to GM1 injections.

The use of biomarkers of target engagement or downstream outcomes are now considered to be important aspects of early clinical development of potential disease-modifying therapeutics, and the use of such biomarkers may improve the likelihood of success of such agents in clinical trials [67]. Currently, there is no biomarker for GM1 and no definitive way at this point to monitor target engagement. These areas need further research and exploration. Several concepts are being explored in the development of biomarkers for GM1 delivery to the brain and for monitoring downstream effects of GM1 and, if successful, these measures will be employed in any future clinical trials of GM1 in PD. Such readouts, if associated with clinical improvements, would provide strong support for the efficacy of GM1 as a disease-modifying therapeutic for PD.
